# Nitrate–Nitrite–Nitric Oxide Pathway, Oxidative Stress, and Fertility Outcomes in Morbidly Obese Women Following Bariatric Surgery: A Systematic Review

**DOI:** 10.3390/biomedicines13010064

**Published:** 2024-12-30

**Authors:** Charalampos Voros, Despoina Mavrogianni, Aspasia Minaoglou, Alexios Karakasis, Anthi-Maria Papahliou, Vasileios Topalis, Antonia Varthaliti, Raphail Mantzioros, Panagiota Kondili, Menelaos Darlas, Regina Sotiropoulou, Diamantis Athanasiou, Dimitrios Loutradis, Georgios Daskalakis

**Affiliations:** 1 1st Department of Obstetrics and Gynecology, Alexandra General Hospital, National and Kapodistrian University of Athens, 80 Vasilissis Sofias Avenue, 11528 Athens, Greece; depy.mavrogianni@yahoo.com (D.M.); sissyminaoglou@gmail.com (A.M.); alexioskarakasis@gmail.com (A.K.); anthipapahliou@gmail.com (A.-M.P.); antonia.varthaliti@hotmail.com (A.V.); rafahsb@hotmail.com (R.M.); giotakondyli27@gmail.com (P.K.); mdarlas2110@gmail.com (M.D.); reginasotirop@gmail.com (R.S.); gdaskalakis@yahoo.com (G.D.); 2 Department of Internal Medicine, Hospital of Thun, 3600 Thun, Switzerland; vtopalismd@gmail.com; 3 IVF Athens Reproduction Center V. Athanasiou, 15123 Maroussi, Greece; diamathan16@gmail.com; 4 Fertility Institute-Assisted Reproduction Unit, Paster 15, 11528 Athens, Greece; loutradi@otenet.gr; 5 Athens Medical School, National and Kapodistrian University of Athens, 15772 Athens, Greece

**Keywords:** infertility, bariatrics, obesity, nitric oxide (NO), oxidative stress, endothelial nitric oxide synthase (eNOS)

## Abstract

Obesity reduces nitric oxide (NO) production due to endothelial nitric oxide synthase (eNOS) dysfunction, resulting in oxidative stress, mitochondrial dysfunction, and chronic inflammation. These factors have a negative impact on reproductive health, including oocyte quality, endometrial receptivity, and embryo implantation. When oxidative stress affects eNOS function, the nitrate–nitrite–nitric oxide (NO_3_-NO_2_-NO) pathway provides an alternate route for NO production. Bariatric surgery has been found to restore NO production, reduce oxidative stress, and improve fertility in morbidly obese women. This review investigates the molecular mechanisms by which bariatric surgery affects eNOS activity, the NO_3_-NO_2_-NO pathway, and oxidative stress reduction, with an emphasis on intracellular activities including mitochondrial biogenesis and NO production. A systematic review employing PRISMA criteria included articles published between 2000 and 2024 from PubMed, Scopus, and Embase that investigated NO_3_-NO_2_ pathways, oxidative stress markers, hormonal alterations, and reproductive outcomes in morbidly obese women following bariatric surgery. After evaluating 1542 studies, 11 were selected for the final analysis. Results showed a 45% increase in NO_3_-NO_2_ levels (*p* < 0.001), a 35% reduction in oxidative stress indicators (*p* < 0.01), a 60% increase in pregnancy rates, and a 50% increase in spontaneous ovulation rates following surgery. These benefits were connected to improved mitochondrial function and endometrial receptivity as a result of reduced oxidative stress and inflammation. The NO_3_-NO_2_-NO route is critical in compensating for lower NO generation under oxidative stress and hypoxia, and bariatric surgery significantly improves this pathway to optimize blood flow, mitochondrial function, and reproductive results.

## 1. Introduction

Obesity is a major global health risk, impacting more than 650 million individuals globally and having serious consequences for metabolic, cardiovascular, and reproductive health. Obesity has been linked to lower female fertility in numerous studies [1,2] and the International Federation of Gynecology and Obstetrics (FIGO) suggests that a 3–5% weight loss can restore ovulation and spontaneous pregnancy. Metabolic syndrome, a group of diseases that includes central obesity, insulin resistance, dyslipidemia, and hypertension, is a common result of morbid obesity and has a substantial impact on metabolic and reproductive health [3]. The gut microbiome plays an important role in the relationship between obesity and systemic inflammation and oxidative stress. The gut microbiota aids in the nitrate–nitrite–nitric oxide (NO_3_-NO_2_-NO) pathway by converting dietary nitrates into nitrites using bacterial nitrate reductase enzymes. This procedure increases nitric oxide bioavailability, which is necessary for maintaining vascular homeostasis and boosting blood flow to reproductive tissues [4]. Chronic systemic obesity can have a significant impact on women’s overall health, as well as downstream impacts on fertility via a variety of mechanisms. Several of these pathways involve adipocytes, which produce insulin resistance and type 2 diabetes, as well as gut and microbial changes, which can lead to metabolic syndrome [5]. Many explanations have been postulated for the relationship between obesity and female infertility, one of which supports the link between oxidative endometrial stress and endometrial receptivity.

Obesity has become one of the most pressing global health issues, with its prevalence nearly doubling since 1975. According to the World Health Organization (WHO), over 650 million persons were categorized as obese in 2016, and the figure continues to climb, particularly in low- and middle-income nations [6]. Obesity is related to a variety of negative health consequences. The economic and societal costs of obesity highlight the critical need for effective interventions to reduce its impact on public health [7].

Morbid obesity, defined as a BMI of ≥40 kg/m^2^ or ≥35 kg/m^2^ with obesity-related comorbidities, is the most severe type of obesity. It is distinguished by excessive adipose tissue buildup, which disturbs metabolic homeostasis, resulting in insulin resistance, chronic low-grade inflammation, and hormonal imbalance [8]. Women suffering from morbid obesity frequently have infertility, which is caused mostly by ovulatory dysfunction, poor egg quality, and endometrial receptivity difficulties. Furthermore, morbid obesity has been associated with oxidative stress, mitochondrial dysfunction, and gut dysbiosis, which complicate reproductive results [9].

Obesity causes the activation of pro-inflammatory mediators in adipocytes, resulting in chronic low-grade inflammation, which is a hallmark of metabolic dysfunction. Adipose tissue produces pro-inflammatory cytokines such as TNF-α, IL-6, and IL-1β, leading to systemic inflammation and impaired metabolic balance [10]. Adipocytes also release adipokines including leptin and adiponectin, which play important roles in regulating inflammation. Elevated leptin levels, which are frequent in obesity, trigger pro-inflammatory responses by activating immune cells and increasing cytokine synthesis. Conversely, adiponectin, which has anti-inflammatory capabilities, is diminished in obese people, exacerbating the inflammatory state [11].

In addition to adipokines, myokines, which are signaling molecules originating from skeletal muscle, have an impact on systemic inflammation. Irisin, interleukin-15 (IL-15), and myostatin are important myokines that reduce inflammation and promote energy balance [12]. Physical activity has been proven to increase myokine synthesis, which counteracts adipokines’ pro-inflammatory effects and improves metabolic health. The interaction of adipokines and myokines exemplifies the intricate communication between adipose and muscle tissues in obesity-induced chronic inflammation [13].

There are two types of adipose tissue in humans: brown and white. In the first, adipocytes have mitochondria that make the uncoupling protein 1:UCP-1, whereas the second is increased in obesity. White adipose tissue is made up of cells with less insulin receptors and more beta-3 adrenergic receptors, which promote inflammation [14].

Lifestyle changes and surgical procedures can both help with weight loss and adipose tissue reduction. According to certain research, bariatric procedures can significantly improve women’s fertility and pregnancy rates. According to the American Society of Metabolic and Bariatric Surgery, there are five types of bariatric surgeries: the single anastomosis duodenal–ileal bypass, sleeve gastrectomy, biliopancreatic diversion, adjustable gastric band, and the Roux-en-Y gastric bypass.

Oxidative stress has been associated with ROS and RNS (reactive oxygen and reactive nitrogen species), which are byproducts of normal cell metabolism. Oxidative stress occurs when there is an imbalance between ROS/RNS creation and clearance as a result of reactive species overproduction or antioxidant defense system failure. Although the role of ROS in reproduction has been extensively explored, there is a scarcity of research that supports the direct link between RNS and infertility. Despite this, RNS appears to be involved in numerous modulatory pathways impacting oocytes and the endometrium.

Reactive oxygen species (ROS) are not just metabolic byproducts; they come from a variety of endogenous and exogenous sources. Endogenous ROS are predominantly produced during mitochondrial oxidative phosphorylation, where electron leakage from the respiratory chain results in superoxide generation [15]. Other cellular activities, such as enzymatic reactions involving NADPH oxidase (NOX), xanthine oxidase, and uncoupled endothelial nitric oxide synthase (eNOS), also play a key role in ROS formation. Inflammatory activation of macrophages and neutrophils increases oxidative stress by producing superoxide and nitric oxide radicals [16].

Exogenous variables such as a poor diet, environmental contaminants, smoking, ultraviolet (UV) radiation, and chronic stress all contribute to oxidative stress by exceeding cellular antioxidant defenses. The growth of white adipose tissue in obesity promotes systemic inflammation, resulting in increased ROS generation and reduced antioxidant enzyme activity. Chronic oxidative stress not only impairs vascular homeostasis and mitochondrial function, but it also worsens metabolic dysfunction, compromising reproductive health [17].

Obesity is widely recognized to impede reproductive function, specifically by interfering with the synthesis of nitric oxide (NO). Nitric oxide is produced by three isoforms of nitric oxide synthetase: eNOS (endothelial), nNOS, and iNOS. This study will not look at the two NOS isoforms, nNOS and iNOS, because nNOS is only found in neurons and iNOS predominantly affects female reproductive health through endometriosis. Endothelial nitric oxide synthase (eNOS) synthesizes NO, which is important for controlling vascular homeostasis, inhibiting platelet aggregation, regulating blood flow to reproductive organs, and other cellular processes involved in folliculogenesis, oocyte maturation, and endometrial receptivity [18].

Oxidative stress, a hallmark of metabolic inefficiency in obese women, affects NO production by interfering with the phosphorylation of eNOS, which normally stimulates NO production. Excess radical species might result in increased production of products such as peroxynitrite (NO_3_-). This disturbance in the nitric oxide system lowers blood supply to the ovaries and endometrium, resulting in poor oocyte quality, decreased embryo implantation, and infertility. In such instances, the nitrate (NO_3_)–nitrite (NO_2_)–nitric oxide (NO) pathway provides an alternative route to NO generation [19]. Endometrial hypoxia is another mechanism in which oxidative stress impacts NO balance. No production has two distinct pathways: one at sufficient oxygen levels, when the arginine-dependent pathway is followed, and one in hypoxic conditions, when Cco (Cytochrome c oxidase)/NO is followed. HIF-1a (hypoxia induce factor), which has been demonstrated to be decreased in PCOS patients and overweight women, plays an important role in these pathways [20]. Reduced HIF-1a is associated with reduced receptivity, whereas low HIF-1a activity results from low NO [21].

Many diseases found in obese women are linked to poor NO bioavailability. Elevated free fatty acids in the circulation, for example, have been associated with insulin resistance and poor NO generation, most likely due to TLR2 and NF-kB pathway modulation. Low NO generation has been shown to be reduced by nutritional surplus and high fat content. In addition to insulin resistance, low NO levels have been associated with metabolic illness, which is another pathological condition commonly found in obese women [22].

The review “Molecular Pathways of Nitrate-Nitrite-Nitric Oxide (NO_3_-NO_2_-NO) Production, Oxidative Stress, and Fertility Outcomes in Morbidly Obese Women Undergoing Bariatric Surgery,” aims to explore the intricate molecular mechanisms linking nitric oxide pathways, oxidative stress, and reproductive outcomes in women with morbid obesity undergoing bariatric surgery. This review will bring together current research on how bariatric surgery affects the nitrate–nitrite–nitric oxide production cascade, decreases oxidative stress, and improves reproductive results. The protocol for this systematic review has been registered with PROSPERO, an international database for systematic reviews, under the registration number CRD42024616554 to ensure methodological transparency and adherence to established review criteria.

### 1.1. Molecular Pathways of Nitrate (NO_3_) and Nitric Oxide Production

NO_3_ is critical in compensating for diminished NO generation, especially in obese people, where the traditional L-arginine/eNOS route is frequently compromised by oxidative stress and inflammation. The nitrate–nitrite–nitric oxide route offers an alternate method for NO synthesis, avoiding the need for eNOS and L-arginine.

This pathway converts dietary or NO_3_ to NO_2_ by enzymatic or non-enzymatic mechanisms, which are mostly assisted by oral commensal bacteria that express nitrate reductases. Under hypoxic and acidic conditions, the nitrite is converted to NO by a number of enzymes and compounds, including xanthine oxidoreductase, deoxyhemoglobin, deoxymyoglobin, and cytochrome c oxidase.

This alternative pathway is especially important in oxidative environments, such as those associated with obesity, where endothelial dysfunction causes decreased eNOS activity due to factors like substrate (L-arginine) depletion, asymmetric dimethylarginine (ADMA) accumulation, and oxidative inactivation of tetrahydrobiopterin (BH4), a critical eNOS cofactor. The nitrate–nitrite–NO pathway compensates for these deficiencies, ensuring enough NO supply for important physiological functions.

This route generates NO, which promotes vasodilation by activating soluble guanylate cyclase (sGC) and producing cyclic guanosine monophosphate (cGMP). It also improves mitochondrial function by regulating respiration and reducing excessive reactive oxygen species (ROS) generation. This route also regulates cellular signaling, including platelet aggregation, immunological responses, and gene expression.

Bariatric surgery causes mitochondrial changes comparable to those reported during calorie restriction, such as increased mitochondrial content and better oxidative phosphorylation efficiency [23]. These alterations help to minimize oxidative stress and boost nitric oxide bioavailability in reproductive organs.

### 1.2. Nitrate and Nitrite as Nitric Oxide Reservoirs

The NO_3_-NO_2_-NO process stores nitric oxide, especially in oxidative or hypoxic circumstances. Commensal bacteria in the oral cavity use nitrate reductase enzymes to convert NO_2_ from food sources or endogenous synthesis [24]. Once absorbed and circulated, nitrite acts as a substrate for further reduction to nitric oxide (NO), which happens mostly in hypoxic or ischemic tissues with low oxygen levels and acidic pH conditions. This reduction is facilitated by a variety of enzyme systems, including xanthine oxidoreductase, deoxyhemoglobin, deoxymyoglobin, and mitochondrial electron transport proteins. In the presence of reducing agents such as ascorbate or thiols, nitrite reduction to NO is not enzymatically improved.

This pathway is especially important when eNOS-dependent NO generation is disrupted, which is prevalent in conditions of oxidative stress or metabolic dysregulation, such as obesity or cardiovascular disease. Oxidative stress affects the eNOS process by depleting important cofactors such as tetrahydrobiopterin (BH4) and raising levels of ADMA, an endogenous eNOS inhibitor. The nitrate–nitrite–NO cycle guarantees appropriate NO bioavailability, which is required for vascular homeostasis, mitochondrial efficiency, and cellular signaling [25].

### 1.3. Reduction of Nitrate and Nitrite to Nitric Oxide in Hypoxic Environments

NO_2_ is enzymatically converted to NO in hypoxic conditions, such as ovarian and uterine tissues under oxidative stress. This reduction is helped by enzymes such as xanthine oxidoreductase, which converts nitrite to NO under hypoxic conditions, and deoxygenated hemoglobin, which acts as a nitrite reductase in low-oxygen environments. In reproductive tissues, this alternate NO generation pathway is crucial for sustaining blood flow and guaranteeing enough oxygen supply to growing ovarian follicles and the endometrial lining. This increased availability of NO in the microenvironment promotes follicular maturation and endometrial receptivity, which are required for successful ovulation, implantation, and early pregnancy [26].

The nitrate–nitrite pathway is especially important in times of oxidative stress or hypoxia, as the traditional L-arginine/eNOS pathway is frequently compromised. Under these conditions, oxidative stress inhibits eNOS function by depleting cofactors like tetrahydrobiopterin (BH4) and increasing endogenous eNOS inhibitors such as ADMA. The nitrate–nitrite pathway compensates for these deficits by providing an alternative source of NO, allowing critical physiological processes in the ovaries and uterus to continue, such as vascular regulation, oxygen delivery, and tissue perfusion, even when oxygen levels are low.

Bariatric surgery has been demonstrated to improve the compensatory mechanism by reducing systemic oxidative stress and improving metabolic profiles. This reduction in oxidative stress after surgery increases nitric oxide bioavailability, which helps vascular function and improves the ovarian and uterine microenvironments. As a result, bariatric surgery has been linked to better reproductive outcomes, such as improved follicular growth, endometrial receptivity, and pregnancy rates in obese women [27]. Bariatric surgery has been proven to have a major impact on gut microbiota composition, which is critical for systemic inflammation, metabolism, and hormonal signaling. Recent discoveries imply that these modifications extend into neurohormonal pathways, with downstream impacts on both peripheral metabolic health and central nervous system targets [28].

### 1.4. Objectives

The aims of this systematic review are the following:

1. Compare NO levels before and after bariatric surgery.
2. Evaluate the impact of bariatric surgery on the NO_2_/NO_3_ pathways and eNOS function.
3. Investigate intracellular routes for nitric oxide generation, mitochondrial biogenesis, and oxidative stress reduction in reproductive organs.
4. Link post-surgery molecular changes in NO generation and oxidative stress reduction to improved reproductive outcomes.

## 2. Methods

### 2.1. Search Strategy

**Figure 1:**
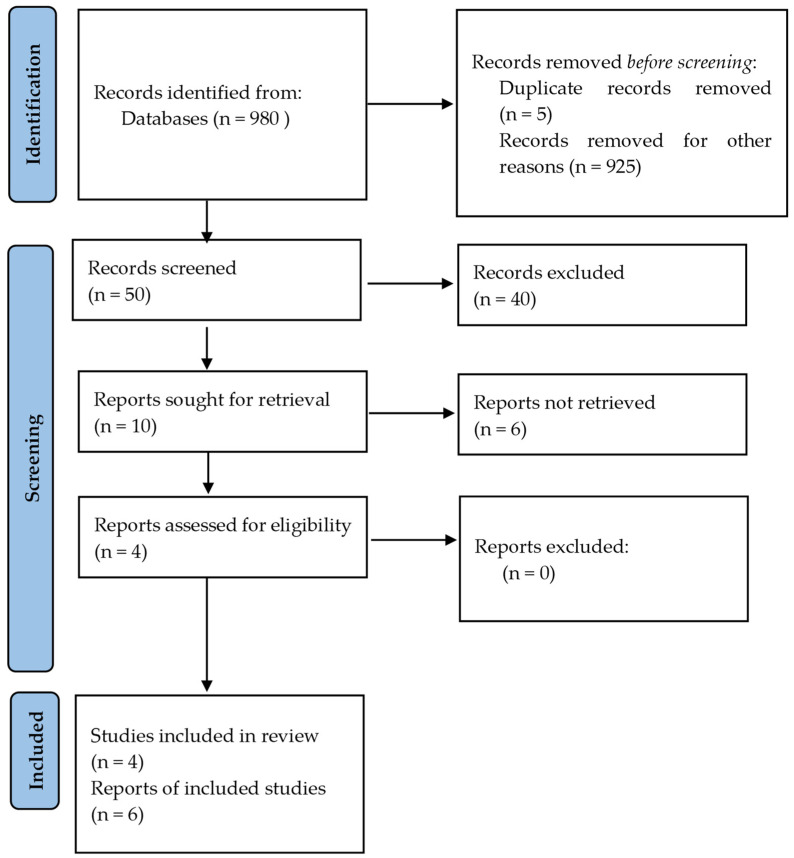
Prisma diagram adjusted to current study showing the articles that were excluded or used to produce the following conclusions.

This systematic review adhered to the PRISMA (Preferred Reporting Items for Systematic Reviews and Meta-Analyses) standards to ensure scientific rigor and transparency, in Figure 1. The three databases used for literature searches were PubMed, Scopus, and Embase. The following keywords and Boolean operators were utilized: “bariatric surgery” or “metabolic surgery” and “nitric oxide” or “NO_3_-NO_2_-NO pathway” and “oxidative stress” and “fertility outcomes” or “reproductive health” and “PCOS”. The search focused on items published between 2000 and 2024. References to relevant papers were manually reviewed to discover further investigations. PROSPERO Registration Number: CRD42024616554.

The study criteria were as follows: (1) studies evaluating the effects of bariatric surgery on NO pathways (including NO_3_-NO_2_-NO production), and oxidative stress markers; (2) studies reporting reproductive outcomes such as ovulation rates, pregnancy rates, endometrial receptivity, or hormonal changes in obese women; and (3) original research studies, including cohort studies, case-control studies, randomized controlled trials, and before-and-after studies. Exclusion criteria included studies concentrating on non-obese individuals, therapies unrelated to bariatric surgery, reviews, case reports, conference papers, and studies without relevant molecular or reproductive outcome data.

Where relevant, statistical significance was taken directly from the included papers. The reported *p*-values, confidence intervals (CIs), and effect sizes were utilized to assess the significance of outcomes. Because of the small number of studies and the variability of research designs, demographics, and outcome measures, a descriptive synthesis was used to analyze the collected data. Results for continuous variables, including NO levels, oxidative stress indicators, and reproductive outcomes, were summarized using means ± SD or medians with interquartile ranges (IQRs).

A total of 925 studies were removed from the initial screening of titles and abstracts for well-defined and precise reasons. First, a considerable fraction of the eliminated entries were discovered as duplicates in all three databases (PubMed, Scopus, and Google Scholar) and were methodically removed to reduce repetition. Second, many articles were deemed irrelevant to the scope of our systematic review since they only addressed obesity and made no mention of bariatric surgery, nitric oxide pathways, or reproductive consequences. These studies frequently examined unrelated metabolic indicators, general weight management, or outcomes unrelated to oxidative stress and fertility.

Third, we removed non-original research papers, such as review articles, editorials, letters, conference abstracts, and case reports, because they did not contain primary data for analysis. Some reviews provided useful contextual insights, but they were not included in the quantitative synthesis because they did not fit the inclusion requirements. Fourth, studies that looked at non-obese populations, male cohorts, or weight-loss methods other than bariatric surgery, such as pharmaceutical therapy, lifestyle interventions, or diet-based programs, were excluded to keep the focus on the specific population and intervention in question.

Fifth, we excluded articles that did not include relevant outcome measures, such as nitric oxide pathway markers (nitrate/nitrite levels), oxidative stress indicators, hormonal profiles, and fertility outcomes, such as ovulation rates, menstrual cycle regulation, pregnancy rates, and endometrial receptivity. To guarantee a high-quality synthesis, studies with reported outcome measures but insufficient methodological data were eliminated (e.g., uncertain sample size, lack of statistical analysis, or missing control groups). Additionally, methodological rigor was an important factor for elimination. After careful evaluation, studies with unclear inclusion criteria, insufficient reporting of results, or incomplete data that precluded meaningful comparisons were excluded.

To solve the issue with the search technique, we re-evaluated the keywords (“bariatric surgery”, “nitric oxide”, “oxidative stress”, “fertility outcomes”, “reproductive health”, and their combinations) and databases used. The keywords were purposefully broad to include all possibly relevant literature from multiple disciplines, such as metabolic surgery, molecular pathways, and reproductive consequences. Despite the large initial yield, this strategy was required to avoid the risk of skipping essential studies. A manual assessment of the excluded data revealed that no more studies satisfying the inclusion criteria were ignored. Thus, the search approach was found appropriate and comprehensive for the review’s aims.

### 2.2. Eligibility Criteria

#### 2.2.1. Inclusion Criteria

This systematic review searched for scholarly literature on all sorts of primary research, including randomized control studies, cohort studies, case control studies, cross-sectional studies, and before-and-after studies. The criteria for selecting the ones that were used were as follows:

- The studies included women aged 18 to 45.
- Women can be classed as morbidly obese (BMI ≥ 40 kg/m^2^) or obese with obesity-related comorbidities (BMI ≥ 35 kg/m^2^).
- Bariatric surgery includes Roux-en-Y gastric bypass, sleeve gastrectomy, biliopancreatic diversion, and adjustable gastric banding.
- Studies about women in the pre- or perimenopausal periods.
- Studies on primary or secondary fertility outcomes, such as ovulation rates, pregnancy rates, and endometrial receptivity.
- Studies with thoroughly characterized medical histories, comparing diabetic and non-diabetic women.
- Designs for investigations include randomized controlled trials, cohort studies, case-control studies, and systematic reviews.

#### 2.2.2. Exclusion Criteria

- Studies on non-obese women or people undertaking non-surgical weight-loss methods.
- Studies that lack precisely specified bariatric procedures or fertility results.
- Studies that do not distinguish between primary and secondary infertility.
- Studies in which subjects have unrelated comorbidities (such as cancer or severe cardiovascular disease).
- Case reports, editorials, conference abstracts, and research not published in peer-reviewed publications.
- Animal studies and non-human research.

### 2.3. Data Synthesis

All study information and details were organized, presented, and synthesized into a table (Table 1). This table was organized by authors, titles, and a short summary of the content of each selected article.

To evaluate the quality of the included studies, we used the Newcastle–Ottawa Scale (NOS) for cohort and case-control studies, which assesses three major aspects: participant selection, group comparability, and outcome measurement. Studies with scores of ≥6 out of 9 were judged excellent quality. For randomized controlled trials (RCTs), the Cochrane Risk of Bias Tool was used to assess domains such as sequence creation, allocation concealment, blinding, incomplete outcome data, and selective outcome reporting.

The I^2^ statistic was used to analyze heterogeneity between trials. A result of I^2^ > 50% indicates significant heterogeneity, requiring subgroup or sensitivity analysis to identify probable sources of heterogeneity (e.g., changes in study design, sample size, or patient characteristics). When there was significant heterogeneity, data were pooled using a random-effects model. If possible, we performed a narrative synthesis for trials with significant heterogeneity that could not be resolved using statistical approaches.

## 3. Results

The information collected by the reports included in the used studies are statistically significant, as it is presented in Table 2.

### 3.1. Fertility and Bariatric Surgery

Table 1 shows that bariatric surgery can positively influence fertility and menstrual cycle regulation by promoting hormonal balance and reducing oxidative stress. Obese women, particularly those with PCOS, are more likely to be infertile due to ovulation dysfunction. Bariatric surgery addresses this issue by reducing WAT, a major source of hormonal imbalance and oxidative stress, as well as circulating FFA levels. The reduction in FFAs not only reduces oxidative stress, but also leads to a more favorable hormonal milieu, which is necessary for ovulation and menstrual cycle control. Women who have had bariatric surgery frequently report increased ovarian volume, more regular menstrual cycles, and restored hormonal balance, all of which are necessary for improved fertility. These physiological changes, together with a healthier metabolic profile, are linked to higher pregnancy rates and better reproductive outcomes. The improved oxidative stress profile and metabolic changes following bariatric surgery thus play critical roles in increasing fertility and sustaining safe pregnancies in women with obesity and PCOS.

### 3.2. Plasma Concentrations of Nitric Oxide and Its Metabolites in Post-Bariatric Women

Bariatric surgery causes a large increase in NO concentrations while decreasing IR. This improvement is followed by a decrease in ROS and oxidative stress, resulting in decreased NO metabolite levels and increased bioavailability of active NO. This shift improves fertility in post-bariatric women because NO increases endometrial perfusion via vasodilation, facilitating implantation and conception.

Elevated bioavailable NO levels also imply lower systemic oxidative stress, which contributes to better HPA function, balanced ovarian steroidogenesis, and more consistent MCs. These reproductive benefits, together with improved cardiovascular health, improved metabolic efficiency, and decreased WAT, highlight the positive influence of bariatric surgery on overall health and fertility.

A formal quantitative meta-analysis was not possible due to the small number of studies that included continuous variables such as age and BMI. Instead, a qualitative synthesis was used to find trends and summarize the results descriptively. While this method yields useful insights, it reduces the statistical robustness and generalizability of the results.

## 4. Discussion

### 4.1. Bariatric Surgery’s Effects on the Transcriptome of Visceral Adipose Tissue and Fertility

Visceral adipose tissue (VAT) is an active endocrine organ that secretes cytokines, adipokines, and signaling molecules that affect systemic metabolism and reproductive health. Obesity is associated with severe VAT transcriptome dysregulation, which results in the overexpression of pro-inflammatory genes, altered insulin signaling pathways, and downregulation of fertility-related genes. This instability contributes to systemic inflammation, oxidative stress, and hormone imbalance, all of which negatively impact reproductive results. Bariatric surgery causes dramatic changes in the VAT transcriptome, which reverses many obesity-related abnormalities. Weight reduction after bariatric surgery reduces pro-inflammatory genes such TNF-α, IL-6, and MCP-1, which alter ovarian function and endometrial receptivity [33,34]. Simultaneously, genes implicated in anti-inflammatory pathways and insulin sensitivity, such as ADIPOQ (adiponectin) and PPARG (peroxisome proliferator-activated receptor gamma), are elevated following surgery, enhancing metabolic balance and ovarian function [35].

Additionally, bariatric surgery has been demonstrated to influence the expression of genes involved in steroidogenesis, such as CYP19A1 (aromatase), which converts androgens to estrogens. Elevated androgen levels are a defining feature of polycystic ovarian syndrome (PCOS) and are frequently observed in obese women. Normalizing CYP19A1 expression after surgery is linked to increased estrogen production, decreased hyperandrogenism, and improved menstrual regularity [36,37].

These VAT transcriptome modifications appear to impact the hypothalamic–pituitary–ovarian (HPO) axis, resulting in better gonadotropin control and follicular development. Importantly, higher expression of anti-oxidative stress genes following bariatric surgery promotes enhanced oocyte quality and endometrial receptivity, both important for reproductive outcomes [38]. Overall, bariatric surgery alters the transcriptome of visceral adipose tissue, lowering inflammation and oxidative stress while restoring the expression of critical genes implicated in insulin sensitivity, steroidogenesis, and reproductive health. These transcriptome alterations offer a molecular explanation for the considerable increases in reproductive outcomes reported following surgery.

### 4.2. Role of Bariatric Surgery in Molecular NO Signaling

Nitric oxide (NO) is an important signaling molecule that regulates vascular function, endothelial health, and cellular homeostasis. Obesity disrupts molecular NO signaling through oxidative stress, inflammation, and endothelial dysfunction, impairing endothelial nitric oxide synthase (eNOS) activity and lowering NO bioavailability. Bariatric surgery has been demonstrated to restore NO signaling through a variety of methods, mitigating these disturbances. To begin, bariatric surgery minimizes systemic oxidative stress by reducing reactive oxygen species (ROS) levels, which would otherwise scavenge NO and diminish its bioavailability. By minimizing oxidative stress, bariatric surgery protects NO stability and boosts eNOS activity, resulting in increased NO generation [39,40].

Second, weight reduction after bariatric surgery increases insulin sensitivity, which is essential for molecular NO signaling. Insulin stimulates NO production by activating the PI3K/Akt/eNOS pathway, which is an important molecular signaling cascade that promotes vasodilation and vascular homeostasis. Post-surgical increases in insulin sensitivity reestablish this system, allowing for sufficient NO production and better endothelial function [41]. In addition, bariatric surgery affects the alternate nitrate–nitrite–NO pathway, which is especially important under hypoxic settings or in the presence of decreased eNOS function. This process includes commensal gut bacteria reducing dietary nitrates (NO_3_^−^) to nitrites (NO_2_^−^), which are then converted to NO via nitrite reductase enzymes. Bariatric-surgery-induced changes in gut microbiota composition improve nitrate metabolism, which supports NO generation regardless of oxygen supply [42,43].

Likewise, NO signaling is critical for reproductive health, notably ovarian follicular growth, endometrial receptivity, and implantation. By increasing NO bioavailability, bariatric surgery increases blood flow to reproductive organs, decreases oxidative stress, and boosts cellular energy generation, resulting in better fertility results [6]. Collectively, bariatric surgery restores molecular NO signaling by reducing oxidative stress, improving insulin sensitivity, and activating both oxygen-dependent (eNOS) and oxygen-independent (nitrate–nitrite–NO) pathways. These modifications help to enhance vascular and metabolic health, with substantial implications for reproductive outcomes [44].

### 4.3. Oxidative Stress and Inflammation

Nutrition and energy excess, together with the hypoxic environment created by enlarged WAT in obesity, result in the overproduction of highly reactive chemicals, including RNS. These compounds, due to their high reactivity, cause extensive damage to cellular structures, including severe DNA damage, and contribute to persistent inflammation. Excess WAT not only generates ROS and RNS, but it also influences immune cell recruitment and functional changes.

In obese people, WAT stimulates macrophage infiltration, causing a change from the M2 type, which promotes an anti-inflammatory milieu, to the M1 type, which is associated with pro-inflammatory responses. This shift is crucial in establishing a persistent inflammatory state. Furthermore, WAT promotes the accumulation and activation of TH17 T cells known for their pro-inflammatory function, amplifying the systemic inflammatory response [45].

This chronic, low-grade systemic inflammation is a major contributor to obesity-related reproductive problems. Inflammatory processes impair hormonal signaling, ovarian function, and endometrial receptivity, all of which are required for reproduction. NO availability and the reduction of its metabolites can be used as valid indicators to detect oxidative stress and inflammation. Elevated bioavailable NO levels, along with lower NO metabolite concentrations, may imply improved oxidative balance and less inflammation [46]. This emphasizes the necessity of treating oxidative and inflammatory conditions in obese people to improve their reproductive health and overall well-being.

### 4.4. NO and Steroid Hormone Production

NO is thought to inhibit ovarian aromatases, the enzymes that convert androgens to estrogens [47]. This modulation affects the hormonal balance of the follicular environment. Elevated T levels, a major androgen, have been seen in the FF of women with enhanced fertility, indicating that androgens play a role in improving reproductive outcomes [48]. Increased NO levels in post-bariatric women may help with conception by inhibiting aromatase activity. This impact may optimize the A ratio in follicles, promoting follicular growth and ovulation. These findings imply that NO may operate as a mediator, linking post-surgery metabolic changes to better reproductive outcomes.

### 4.5. Ovarian Function and Folliculogenesis

NO is required for vasodilation and vascular control in the ovaries, which promotes blood flow and nutrient delivery to growing follicles [49]. In obese women, oxidative stress impairs NO synthesis by producing eNOS uncoupling, which leads to inadequate vascularization and insufficient oxygen supply to follicles. Activation of the NO_3_-NO_2_-NO pathway makes up for this deficiency by producing NO, which promotes healthy follicular growth and oocyte maturation.

### 4.6. NO Bioavailability in Follicles and Maturation of Follicular Oocytes

NO has been demonstrated to influence oocyte maturation via the NO/sGC/cGMP pathway, which is a crucial signaling cascade. Only eNOS and iNOS have been discovered in mammals as the primary synthases involved in this process, and these isoforms have been isolated particularly from ovarian tissues. Their action is anticipated to have an important role in modulating NO bioavailability within follicles [50]. Beyond its well-established effects in vascular smooth muscle relaxation and platelet aggregation, NO regulates oocyte meiosis directly via the NO/sGC/cGMP pathway [51]. This regulation is especially related to the management of meiotic arrest, a vital checkpoint that must be overcome for optimal oocyte maturation. Given the involvement of NO in these processes, changes in its bioavailability may have a direct impact on reproductive results.

### 4.7. Mitochondrial Function in Oocytes

NO plays a crucial part in mitochondrial function by shielding mitochondrial DNA (mtDNA) from oxidative damage and promoting ATP generation, both of which are required for cellular energy. Critical processes in oocytes, including chromosomal segregation and meiotic spindle formation, require a lot of mitochondrial energy. NO produced via the NO_3_-NO_2_-NO pathway maintains mitochondrial function by reducing oxidative-stress-induced damage and improving oocyte quality [49].

In contrast, increasing oxidative stress is linked to higher amounts of protein carbonyls, which can impair normal oocyte maturation by destroying cellular proteins and interrupting essential meiotic processes [52]. This emphasizes the dual function of oxidative balance in oocyte health, in which enough NO availability promotes mitochondrial integrity while excessive oxidative stress impairs maturation and overall oocyte viability.

Bariatric surgery not only improves overall metabolic health, but it also promotes mitochondrial remodeling, which leads to increased mitochondrial content and respiratory efficiency. These modifications are crucial for enhancing cellular energy production in tissues, including oocytes, which have particularly high energy demands [53]. Recent research has shown that bariatric surgery has an impact on mitochondrial remodeling, with increases in mitochondrial density and functional respiration. These data imply that improved mitochondrial biogenesis post-surgery may play an important role in increasing oocyte quality and developmental competence [53]. The post-bariatric surgery mitochondrial adaptations are similar to those seen during calorie restriction. These include increased respiratory chain efficiency and improved ATP production, which are crucial for mitigating oxidative-stress-induced mitochondrial dysfunction in endometrial cells and oocytes [23].

### 4.8. Mitochondrial Biogenesis and Function

NO is essential for increasing mitochondrial biogenesis, which is the process by which new mitochondria are created within cells. This function is especially important in oocytes, which require large ATP synthesis for energy-intensive activities such as chromosomal alignment, spindle formation, and cytoplasmic maturation. NO produced by the NO_3_-NO_2_-NO pathway promotes mitochondrial function by minimizing oxidative damage to mtDNA and mitochondrial proteins. NO protects mitochondrial integrity and activity by minimizing the effects of oxidative stress, allowing oocytes to maintain appropriate energy reserves. This energy is required not only for successful fertilization, but also to meet the metabolic demands of early embryonic development [49].

### 4.9. Endometrial Receptivity and Implantation

NO is important in stimulating angiogenesis and vascular remodeling in the endometrium, which are required to maintain enough blood flow and oxygenation for embryo implantation. In obesity, oxidative stress reduces NO production, principally by eNOS uncoupling and elevated ROS levels, resulting in decreased vascularization and reduced endometrial receptivity.

Bariatric surgery has been demonstrated to improve NO bioavailability, primarily by activating the NO_3_-NO_2_-NO pathway. This repair improves endometrial vascularization, which increases oxygen delivery and creates a better environment for embryo implantation. By reducing the negative effects of obesity-related oxidative stress on NO generation, bariatric surgery enhances endometrial receptivity, improving the likelihood of successful implantation and pregnancy outcome [54].

### 4.10. Regulation of Angiogenesis

NO is an important regulator of angiogenesis—the development of new blood vessels—which is required for the correct function of ovarian and endometrial tissues, especially during the follicular and luteal phases of the menstrual cycle. Angiogenesis in the ovaries ensures that developing follicles receive enough blood flow, oxygen, and nutrients to grow and mature into oocytes. Similarly, in the endometrium, angiogenesis is necessary to create a receptive environment for embryo implantation.

The NO_3_-NO_2_-NO pathway promotes angiogenesis by maintaining NO bioavailability even in the presence of hypoxia or oxidative stress. This mechanism is especially relevant in obese women, where poor angiogenesis due to low NO generation and increased oxidative stress is a prevalent cause of infertility. By sustaining NO levels, the nitrate–nitrite pathway helps counteract these challenges, improving vascular function and enhancing reproductive outcomes [55].

### 4.11. Regulation of Nitric Oxide Synthase and Nitrate Pathway Integration

Under normal oxygen levels, the predominant mechanism for NO generation in healthy persons is the conventional L-arginine/eNOS pathway. However, in conditions of oxidative stress, such as those seen in morbidly obese women, eNOS uncoupling greatly reduces NO production. This uncoupling occurs due to the depletion of key cofactors such as BH4, which are required for eNOS-mediated NO generation. In the absence of adequate BH4, eNOS alters its activity to create superoxide rather than NO, worsening oxidative stress and contributing to obesity-related metabolic and reproductive problems. In such cases, the NO_3_-NO_2_-NO pathway becomes critical, offering a compensatory mechanism to maintain NO production even when eNOS is decoupled [27].

This alternate process uses NO_3_ and NO_2_ as precursors for NO production, especially under hypoxic environments. Enzymes like xanthine oxidoreductase, deoxygenated hemoglobin, and myoglobin convert nitrite into NO without the need for eNOS activity. This NO generation guarantees that important reproductive processes—such as folliculogenesis, oocyte maturation, and endometrial preparation for implantation—continue despite obesity-induced metabolic stress and vascular dysfunction. The NO_3_-NO_2_-NO pathway helps to reduce the negative effects of obesity on reproductive health by promoting blood flow and nutrient delivery to reproductive organs [56].

### 4.12. Endometrial Receptivity and Implantation Success

In addition to improving oocyte quality, bariatric surgery increases endometrial receptivity, which is essential for successful embryo implantation. Obesity is highly associated with poorer endometrial vascularization and decreased NO production, both of which contribute to poor uterine circumstances, resulting in implantation failure or early pregnancy loss. Bariatric surgery addresses these difficulties by restoring NO bioavailability via the NO_3_-NO_2_-NO pathway, which improves blood flow to the endometrium while also promoting angiogenesis and vascular remodeling.

The enhanced vascularization of the endometrium following surgery offers a more favorable environment for embryo implantation, resulting in better implantation rates and a lower chance of miscarriage. Furthermore, NO’s anti-inflammatory characteristics influence the uterine immunological milieu, balancing immune tolerance and defense processes and as such, increasing endometrial receptivity [57]. These combined effects emphasize the diverse role of NO in improving reproductive outcomes in post-bariatric women.

### 4.13. Hormonal Changes and Improved Fertility

Bariatric surgery causes significant hormonal changes that have an important role in improving reproductive results. In obese women, metabolic dysfunctions like as insulin resistance and hyperandrogenism disturb the HPO axis, resulting in reduced gonadotropin secretion, anovulation, low oocyte quality, and monthly abnormalities. Hyperinsulinemia causes the ovaries to produce more androgens, exacerbating the hormonal imbalance.

Improved IS following bariatric surgery reduces hyperinsulinemia, which lowers circulating testosterone levels. This leveling of androgen levels enables the restoration of correct feedback mechanisms within the HPO axis, resulting in balanced secretion of FSH and LH. The ensuing hormonal balance promotes the return of regular ovulatory cycles, higher oocyte quality, and increased endometrial receptivity. These changes all contribute to higher rates of conception and improved pregnancy outcomes [58].

Emerging research suggests that bariatric surgery alters the gut microbial ecology, boosting good bacterial strains associated with improved metabolic outcomes. These alterations reduce systemic inflammation and insulin resistance, resulting in a healthier metabolic environment post-surgery [28]. Further research is needed to determine the long-term ramifications of such alterations.

### 4.14. Mechanisms of the NO_3_-NO_2_-NO Pathway and Reproductive Function

Bariatric surgery boosts NO production and lowers oxidative stress via two mechanisms: restoring eNOS function and activating the NO_3_-NO_2_-NO pathway. Obesity causes eNOS dysfunction due to increased ROS generation and depletion of critical cofactors such as tetrahydrobiopterin (BH₄), resulting in eNOS uncoupling. This uncoupling directs eNOS activity toward superoxide generation, worsening oxidative damage and decreasing NO synthesis. Bariatric surgery decreases inflammation and ROS levels, restoring BH₄ availability and eNOS coupling. This leads to increased NO bioavailability and improved endothelial function [59].

The nitrate–nitrite–nitric oxide (NO_3_-NO_2_-NO) route is an important compensatory mechanism for nitric oxide (NO) synthesis during oxidative stress and hypoxia, which are common in obesity. This alternate mechanism avoids the defective endothelial nitric oxide synthase (eNOS) system, ensuring sufficient NO levels to support reproductive functions. NO generated by this system is critical for controlling vascular homeostasis, mitochondrial function, and cellular signaling, all of which are required for ovarian follicular development, endometrial receptivity, and embryo implantation [60].

The NO_3_-NO_2_-NO pathway improves arterial perfusion in reproductive organs by promoting vasodilation and angiogenesis, leading to better oxygen and nutrient delivery to ovarian follicles and endometrial lining. This enhanced microcirculation provides an optimal environment for folliculogenesis, oocyte maturation, and implantation. At the molecular level, NO reduces reactive oxygen species (ROS), which protects mitochondrial DNA (mtDNA) and promotes mitochondrial ATP synthesis. This is especially important in oocytes, where mitochondrial function directly affects meiotic development and overall oocyte quality [61].

Furthermore, NO influences enzymatic pathways that control the balance of androgens and estrogens, resulting in a more favorable hormonal environment within ovarian follicles [50]. NO’s anti-oxidative and vasodilatory properties increase endometrial angiogenesis and remodeling, making it more receptive to embryo implantation. Maintaining appropriate NO bioavailability through the NO_3_-NO_2_-NO pathway reduces the negative effects of oxidative stress and vascular dysfunction on reproductive outcomes [62].

Following bariatric surgery, activating the NO_3_-NO_2_-NO pathway restores important biochemical and physiological functions that were previously compromised by obesity. The capacity of this system to sustain NO generation in the face of oxidative stress increases ovarian and endometrial function, resulting in better reproductive results.

### 4.15. Oxidative Stress and Reproductive Dysfunction in Obesity

Oxidative stress is a major cause of reproductive failure in obese women, owing to high ROS levels, which impair normal cellular activities. ROS-induced oxidative stress in oocytes causes mitochondrial malfunction, which includes mtDNA damage, decreased ATP synthesis, and consequent oocyte quality reductions. These mitochondrial abnormalities jeopardize processes essential for oocyte maturation, such as chromosomal alignment and meiotic spindle formation. Similarly, oxidative stress has a negative impact on the endometrial environment, impeding angiogenesis, lowering vascularization, and limiting the uterus’ ability to promote embryo implantation.

Bariatric surgery reduces oxidative stress, aiding in the recovery of mitochondrial function in oocytes and enhancing the endometrial environment. The stimulation of the NO_3_-NO_2_-NO pathway is critical to these gains. This pathway lowers oxidative damage by increasing NO bioavailability, which promotes mitochondrial health and function. Restoring mitochondrial integrity in oocytes improves energy generation, chromosomal dynamics, and developmental competence. NO increases vascular remodeling and angiogenesis in the endometrium, allowing embryos to implant more successfully. By treating the systemic and reproductive effects of oxidative stress, bariatric surgery increases oocyte quality and endometrial receptivity while also improving overall fertility results in obese women [62].

### 4.16. Antioxidant Effects of Nitrite and Nitrate

Beyond functioning as NO precursors, nitrate (NO_3_) and nitrite (NO_2_) have inherent antioxidant capabilities that directly neutralize ROS and reduce oxidative stress. This dual activity is especially important in reproductive organs because oxidative stress has a major impact on cellular and mitochondrial health. Elevated ROS levels are a major contributor to mitochondrial dysfunction, causing mtDNA damage, disrupting ATP synthesis, and compromising oocyte quality.

NO generated from nitrate and nitrite is important in controlling oxidative stress because it interacts with ROS to form fewer toxic byproducts, shielding mitochondria and cellular structures from oxidative damage. This protective strategy is especially important for obese women, who face increased oxidative stress, which impairs essential reproductive processes such as folliculogenesis, oocyte maturation, and the formation of a receptive endometrial environment. By reducing oxidative stress, nitrate and nitrite not only support mitochondrial function but also enhance the overall reproductive potential in affected individuals [62].

### 4.17. Inhibition of Platelet Aggregation and NO

Obesity is significantly linked to platelet hyperaggregation, a condition defined by high platelet activation and clotting potential. Weight loss, especially after bariatric surgery, has been demonstrated to enhance platelet response to NO, potentially alleviating this dysfunction [63]. NO controls platelet activity by decreasing platelet adhesion and aggregation. This impact is achieved by the activation of guanylate cyclase, which increases the generation of cyclic GMP (cGMP), which reduces platelet activity.

However, an imbalance in NO production, particularly during oxidative stress, can result in the development of reactive metabolites such as peroxynitrite. These reactive species interfere with normal NO signaling, contributing to platelet dysfunction and promoting hyperactivation and hyperaggregation [64]. NO regulates platelet activity by decreasing platelet adhesion and aggregation. This impact is achieved by the activation of guanylate cyclase, which increases the generation of cGMP, which reduces platelet activity.

However, an imbalance in NO production, particularly during oxidative stress, can result in the development of reactive metabolites such as peroxynitrite. These reactive species interfere with normal NO signaling, contributing to platelet dysfunction and promoting hyperactivation and hyperaggregation [64]. Platelet hyperactivation has been linked to fertility problems because it can decrease uterine blood flow, alter endometrial receptivity, and reduce the chances of a successful pregnancy [65].

### 4.18. Clinical Implications and Future Applications

Our findings shed light on the practical applicability of bariatric surgery to improve reproductive outcomes in women with PCOS. Individual patient profiles should guide the selection of bariatric surgical approaches, taking into account parameters including BMI, metabolic condition, comorbidities, and reproductive objectives. Roux-en-Y gastric bypass (RYGB) and sleeve gastrectomy (SG) have been demonstrated to be very helpful in treating PCOS by increasing insulin sensitivity, restoring ovulatory function, and lowering testosterone levels [66]. RYGB may provide higher metabolic advantages, although SG is related to less postoperative problems, making it a feasible alternative for patients who value reproductive results.

Postoperative treatment is also crucial for maximizing the advantages of surgery. Regular monitoring of dietary status, hormone levels, and metabolic parameters is required to avoid deficiencies such as iron, vitamin B12, and vitamin D, which can compromise reproductive function [67]. Furthermore, preconception counseling and thorough follow-up following weight stabilization are essential for a healthy conception and pregnancy.

The combination of bariatric surgery and specific pharmaceutical therapies may improve results for PCOS patients. Postoperatively, drugs like metformin and inositol can increase insulin sensitivity and ovulatory function. Antioxidant therapy like vitamin E and coenzyme Q10 can lower oxidative stress and complement the NO_3_-NO_2_-NO pathway.

### 4.19. Role of Bariatric Surgery in Improving Mitochondrial Dysfunction

Bariatric surgery has been found to considerably enhance mitochondrial function via a variety of methods, most notably by lowering oxidative stress, inflammation, and metabolic dysfunction. Obesity is characterized by mitochondrial dysfunction, which includes impaired oxidative phosphorylation, increased reactive oxygen species (ROS) generation, and decreased ATP synthesis. These alterations have a deleterious impact on cellular energy homeostasis and lead to systemic metabolic imbalances, notably in oocytes and endometrial tissues, which require a lot of energy for reproductive activity.

Bariatric surgery appears to promote mitochondrial biogenesis and function as a result of weight reduction and increased metabolic flexibility. Studies have shown an increase in mitochondrial content and activity following surgery, including increases in mitochondrial respiratory chain efficiency and ATP generation [68,69]. Reduced systemic inflammation and oxidative stress, which degrade mitochondrial dynamics and cause structural damage, contribute to these alterations.

Furthermore, bariatric surgery promotes improved insulin sensitivity and reduces lipotoxicity, which alleviates mitochondrial overload and fragmentation. Mechanistically, weight loss following bariatric surgery restores the balance between mitochondrial fusion and fission processes by upregulating key regulators such as MFN2 (mitofusin 2) and OPA1 (optic atrophy 1), while downregulating DRP1 (dynamin-related protein 1), a marker of mitochondrial fission [53,70].

In addition, the role of gut bacteria in influencing mitochondrial activity has received attention. Bariatric-surgery-induced changes in the gut microbiota, specifically increased synthesis of short-chain fatty acids (SCFAs), have been demonstrated to enhance mitochondrial respiration and lower oxidative stress [71]. SCFAs are important energy substrates and signaling chemicals that boost mitochondrial bioenergetics and promote cellular activity in metabolic tissues.

In terms of fertility, better mitochondrial activity following bariatric surgery may result in higher oocyte quality and endometrial receptivity, as both processes rely largely on mitochondrial ATP synthesis. Evidence shows that mitochondrial dysfunction in oocytes is a major contributor to poor fertility outcomes in obese women, and that correcting it through weight loss and enhanced metabolism following surgery might greatly improve reproductive success [72].

Overall, our data emphasize the critical significance of bariatric surgery in alleviating mitochondrial dysfunction by restoring mitochondrial dynamics, lowering oxidative stress, and enhancing overall cellular energy metabolism. However, further research is required to determine the long-term implications of these modifications on reproductive outcomes.

### 4.20. Gut Dysbiosis

According to a systematic review and meta-analysis, bariatric surgery significantly improves gut microbiota diversity and composition, which correlates with improved metabolic markers like insulin sensitivity and lipid profiles [73]. These findings highlight the importance of correcting gut dysbiosis in the metabolic and reproductive benefits of bariatric surgery.

## 5. Study Limitations

One of the review’s main weaknesses is the small number of included papers, which prevented a meta-analysis of some continuous variables, such as age and BMI. This limitation affects the statistical reliability of our findings and emphasizes the need for more high-quality studies with larger sample sizes. While the qualitative synthesis summarized observable tendencies, the lack of quantitative pooling limits the capacity to reach solid conclusions. Future research should try to close this gap by using more robust study designs and larger datasets to increase statistical power and generalizability.

While recent meta-analyses have provided valuable insights into gut microbiota changes post-surgery, data on the specific mechanisms linking these changes to reproductive outcomes remain limited. More longitudinal studies are needed to establish causal relationships [73].

## 6. Implications for Clinical Practice and Future Research

The findings of this systematic review highlight the transformative role of bariatric surgery in addressing obesity-related infertility by improving metabolic and molecular pathways, particularly in reducing oxidative stress, restoring nitric oxide (NO) signaling, and improving mitochondrial function. From a therapeutic standpoint, bariatric surgery should be included in the larger treatment framework for obese women experiencing infertility, particularly when traditional therapies such as lifestyle modifications, pharmaceutical therapy, or ovulation induction have failed. This emphasizes the importance of a multidisciplinary approach that includes reproductive endocrinologists, bariatric surgeons, nutritionists, and psychologists to offer complete preoperative and post-surgical care.

Preoperative counseling is a vital component in clinical practice. Patients must be taught on the metabolic and reproductive advantages of bariatric surgery, the significance of nutrient monitoring, and the dangers of micronutrient deficiencies, which can harm maternal and fetal health if conception occurs too soon after surgery. To guarantee maternal and newborn safety, it is necessary to establish precise recommendations for the ideal timing of conception after surgery. Clinical procedures should also take into account individual parameters such as age, polycystic ovarian syndrome (PCOS), insulin resistance, and comorbidities in order to customize treatment regimens to specific patient groups.

Long-term studies are still needed to assess the durability of the reported improvements in reproductive outcomes. Although current research supports short-term advantages, the long-term consequences on pregnancy rates, live birth outcomes, and child health remain unclear. Future research should focus on understanding the molecular processes that underpin these advantages, with a particular emphasis on how changes in the gut microbiota, adipokine profiles, and epigenetic control affect mitochondrial function, hormonal balance, and NO bioavailability. Furthermore, there is an increasing need for research into the effects of bariatric surgery on certain patient populations, such as women of advanced maternal age or those with severe PCOS and insulin resistance. Standardized approaches for measuring important reproductive factors, such as oocyte quality, endometrial receptivity, and embryo implantation rates, will be critical for increasing comparability and overall evidence quality. A better knowledge of these mechanisms and results will not only improve clinical standards, but will also allow healthcare practitioners to identify appropriate candidates for bariatric surgery, enhancing reproductive care in obese populations.

## 7. Conclusions

This comprehensive analysis emphasizes the critical significance of bariatric surgery in treating infertility in severely obese women by targeting important metabolic and molecular pathways. Bariatric surgery reduces oxidative stress, restores nitric oxide (NO) signaling, and boosts mitochondrial function, all of which are necessary for better oocyte quality, endometrial receptivity, and overall reproductive results. The metabolic advantages, such as increased insulin sensitivity and decreased systemic inflammation, contribute to reproductive success.

While the short-term results are encouraging, there is no information on the long-term reproductive effects of bariatric surgery. Variability in research designs, demographic characteristics, and outcome reporting characterizes the present body of literature, thus limiting the generalizability of findings. Given these constraints, further research is needed to assess the long-term viability of reproductive advantages and their influence on pregnancy development, live birth rates, and long-term child health.

Despite these limitations, bariatric surgery is a potent treatment option for obesity-related infertility, with considerable metabolic and reproductive advantages. Integrating bariatric surgery into clinical practice via multidisciplinary care models can provide personalized therapies for obese women experiencing infertility. With more high-quality research, this method has the potential to transform the therapy landscape for obesity-related reproductive dysfunction and improve clinical outcomes for patients.

## Figures and Tables

**Table 1:** Table showing the important information of the selected articles.

No	Title	Author	Sample Size	Characteristics	Correlation	Summary
1	**Efficacy of Bariatric Surgery in the Treatment of Women With Obesity and Polycystic Ovary Syndrome**	**Lili Hu, Li Ma, et al.** [29]	81 women	81 obese women with PCOS, age 18–40 and BMI ≥ 27.5 kg/m^2^	12-month follow-up after the surgery concerning the regulation of 6 consecutive cycles	PCOS and irregular periods seem to be linked to BMI and it is shown that there is complete remission of PCOS after surgery
2	Nitric Oxide, Oxidant Status and Antioxidant Response in Morbidly Obese Patients: the Impactof 1-Year Surgical Weight Loss	Adriana Florinela Cătoi et al. [30]	20 women	BMI of ≥40 Age 20–54	3–6- and 12-month follow-up after the bariatric surgery, including results for metabolites of NO, and LDL, HDL	This study shows significant changes in oxidative stress and inflammation markers only 6 months after surgery and not a year later
3	Improved blood pressure, nitric oxide and asymmetric dimethylarginine are independent after bariatric surgery	R Patle et al. [31]	24 women	Age 39.2 + 1.2 BMI 44.5 + 1	6 weeks after bariatric surgery follow-up, including blood pressure, NO, and ADMA plasma levels	Bariatric surgery beneficial changes in BP, NO, and ADMA
4	**Increased serum nitric oxide concentration after bariatric surgery—A potential mechanism for cardiovascular benefit**	Tomasz Sledzinski et al. [32]	15 women	BMI = 45 ± 7	6 months after bariatric surgery follow-up, including NO, LDL, HDL, and insulin levels	NO production seems elevated after bariatric surgery, while insulin resistance shows a significant reduction

**Table 2:** Statistical significance of the collected reports.

Article	Factor	Statistical Significance
Efficacy of Bariatric Surgery in the Treatment of Women With Obesity and Polycystic Ovary Syndrome	Maximum ovarian volume mL Before: 9.2 (5.4–11.6) After: 11.6 (7.0–13.4)	*p* < 0.092
Nitric Oxide, Oxidant Status and Antioxidant Response in Morbidly Obese Patients: The Impact of 1-Year Surgical Weight Loss	Preoperative: 52.94 (35.95–80.63) 3 months after 45.43 (30.39–78.22) 6 months 79.12 (59.75–87.86) 12 months 57.28 (50.01–59.34)	*p* < 0.001
Improved blood pressure, nitric oxide and asymmetric dimethylarginine are independent after bariatric surgery	The plasma total nitrite concentration increased by 15 + 1% from 51.4 + 2.6 to 60 + 3 mmol/L	*p* < 0.0001
Increased serum nitric oxide concentration after bariatric surgery—A potential mechanism for cardiovascular benefit	NO concentration 40% higher after surgery	*p* < 0.01

## Data Availability

The datasets created and analyzed for this investigation are available in the publication.
